# Radiological feature heterogeneity supports etiological diversity among patient groups in Meniere’s disease

**DOI:** 10.1038/s41598-023-36479-5

**Published:** 2023-06-26

**Authors:** David Bächinger, Noemi Filidoro, Marc Naville, Norman Juchler, Vartan Kurtcuoglu, Joseph B. Nadol, Bernhard Schuknecht, Tobias Kleinjung, Dorothe Veraguth, Andreas H. Eckhard

**Affiliations:** 1 Department of Otorhinolaryngology, Head and Neck Surgery, University Hospital Zurich, Zurich, Switzerland; 2 University of Zurich, Zurich, Switzerland; 3 Institute of Applied Simulation, ZHAW University of Applied Sciences, Wädenswil, Switzerland; 4 The Interface Group, Institute of Physiology, University of Zurich, Zurich, Switzerland; 5 Otopathology Laboratory, Massachusetts Eye and Ear, 243 Charles Street, Boston, MA 02114 USA; 6 Department of Otolaryngology - Head & Neck Surgery, Harvard Medical School, Boston, MA USA; 7 Medical Radiological Institute MRI, Zurich, Switzerland

**Keywords:** Inner ear, Neurological disorders, Magnetic resonance imaging

## Abstract

We aimed to determine the prevalence of radiological temporal bone features that in previous studies showed only a weak or an inconsistent association with the clinical diagnosis of Meniere’s disease (MD), in two groups of MD patients (n = 71) with previously established distinct endolymphatic sac pathologies; i.e. the group MD-dg (ES degeneration) and the group MD-hp (ES hypoplasia). Delayed gadolinium-enhanced MRI and high-resolution CT data were used to determine and compare between and within (affected vs. non-affected side) groups geometric temporal bone features (lengths, widths, contours), air cell tract volume, height of the jugular bulb, sigmoid sinus width, and MRI signal intensity alterations of the ES. Temporal bone features with significant intergroup differences were the retrolabyrinthine bone thickness (1.04 ± 0.69 mm, MD-hp; 3.1 ± 1.9 mm, MD-dg; p < 0.0001); posterior contour tortuosity (mean arch-to-chord ratio 1.019 ± 0.013, MD-hp; 1.096 ± 0.038, MD-dg; p < 0.0001); and the pneumatized volume (1.37 [0.86] cm^3^, MD-hp; 5.25 [3.45] cm^3^, MD-dg; p = 0.03). Features with differences between the affected and non-affected sides within the MD-dg group were the sigmoid sinus width (6.5 ± 1.7 mm, affected; 7.6 ± 2.1 mm, non-affected; p = 0.04) and the MRI signal intensity of the endolymphatic sac (median signal intensity, affected vs. unaffected side, 0.59 [IQR 0.31–0.89]). Radiological temporal bone features known to be only weakly or inconsistently associated with the clinical diagnosis MD, are highly prevalent in either of two MD patient groups. These results support the existence of diverse—developmental and degenerative—disease etiologies manifesting with distinct radiological temporal bone abnormalities.

## Introduction

Meniere’s disease (MD) is believed to be an inner ear disorder with diverse, yet unknown, etiologies^1–3^. Although a plethora of temporal bone (TB) abnormalities has been described in MD patients, including, amongst others, poor pneumatization and arrested growth of several bone features, as well as vascular anomalies of the sigmoid sinus (Ss) and the jugular bulb (JB), these features were either highly variably expressed between individual patients or inconsistently prevalent among patient cohorts^4–8^; thus, drawing their pathogenic significance into question. Recently emerging genotypes^9–11^, endotypes^12,13^, and clinical phenotypes^14,15^ in MD begin to delineate more homogenous patient groups, eventually opening up new opportunities to investigate the long-suspected diverse disease etiologies. ‘Endotypes’ refers to disease subtypes defined by specific biological mechanisms that lead to the same clinical manifestation^16^. Considering these ongoing advances, it appears it is worth revisiting some of the previously reported TB features under the new hypothesis that each feature is associated with a distinct disease etiology and thus, is prevalent only in a subset of MD patients. Using CT and MRI data, we divided a retrospective cohort of MD patients into two previously defined groups with suspected different disease etiologies, as indicated by their different histopathologies of the endolymphatic sac (ES)^12^ and the distinguishing radiological (CT/MRI) surrogate markers for both ES pathologies^13^. Accordingly, patients either (1) had developmental hypoplasia of the ES as indicated by the radiological surrogate marker—the angular trajectory of the bony vestibular aqueduct being ≥ 140°—and were assigned to the “MD-hp group”, or (2) had progressive degeneration of the ES as indicated by the radiological angular trajectory of the bony vestibular aqueduct being ≤ 120°, and were assigned to the “MD-dg group”^12,13,15^. In this study we then compared between groups as well as between clinically affected and non-affected TBs within groups, radiological TB features, including geometrical features (contours and dimensions), the volume of the air cell tracts, the height of the jugular bulb, the width of the sigmoid sinus, and Gd-MR signal intensity alterations of the ES. Some of the geometrical features were cross-examined in histologically processed temporal bones from deceased MD-hp and MD-dg patients. We aimed to identify radiological features that would further support the existence of etiologically distinct patient groups, and which could be used as additional clinical markers to distinguish MD-dg and MD-hp patients.

## Materials and methods

### Study population

We included a retrospective cohort (n = 71) of patients with definite uni- or bilateral MD^17^, for whom high-resolution CT (HRCT) and/or gadolinium-enhanced MRI (Gd-MRI) data including a delayed Gd-enhanced 3D-IR sequence of the TBs were available. This cohort was already described in another recent retrospective study^15^. The cohort characteristics are presented in Table 1.

**Table 1:** Key demographic characteristics of the study cohort.

	Total MD patients (n = 71)	MD-dg (n = 55)	MD-hp (n = 16)
Age at inclusion	56.8 (12.7) years	57.8 (13.6) years	53.6 (8.2) years
Female to male ratio	31 (43.7%):40 (56.3%)	30 (54.5%):25 (45.5%)	1 (6.2%):15 (93.8%)
Unilateral:bilateral ratio	64 (90.1%):7 (9.9%)	52 (94.5%):3 (5.5%)	12 (75.0%):4 (25.0%)
Disease duration	10.1 (6.1) years	9.9 (6.4) years	10.9 (4.9) years

### Patient group assignment

Patients were assigned either to the MD-hp or the MD-dg group according to a previously described method in which the angular trajectory of the vestibular aqueduct (ATVA) in the axial imaging plane serves as an indicator for ES hypoplasia (angle ≥ 140°) and ES degeneration (angle ≤ 120°), respectively^13^. Patients with Meniere’s syndrome secondary to other disease processes^18^ were not included in this cohort.

### Archival human TB specimens

To investigate the true (histological) thickness of the bony covering of the posterior semicircular canal, 33 specimens from cases with a clinical diagnosis of definite MD and 30 specimens from cases with normal age-related audiometric threshold patterns and no history of otologic disease, all processed for light microscopic studies^18^, were used in this study. Among the specimens from deceased MD patients, the ES was histologically determined as either hypoplastic, and the individual specimen was assigned to the MD-hp subgroup (n = 15), or as degenerated, and the individual specimen was assigned to the MD-dg subgroup (n = 18)^12^.

### Clinical imaging

All patients underwent 3 T MRI using a 64-channel head coil of the TBs to rule out intra- or retrocochlear pathologies and to detect endolymphatic hydrops. Using previously published protocols, 4 h after intravenous contrast administration (Gadovist; Bayer-Schering Pharma, Berlin, Germany; 1.0 mmol/mL at a dose of 0.2 mmol/kg), a 3D real inversion recovery sequence was acquired using the following parameters: field of view of 190 mm, section thickness of 0.8 mm, matrix size 384 × 384, repetition time of 6000 ms, echo time of 177 ms, inversion time of 2000 ms, flip angle of 180°, bandwidth of 213 Hz/pixel, and scan time of 15 min^19,20^.

Dedicated TB HRCT imaging was available for a subset of patients (n = 12). HRCT data were reconstructed separately for each TB in the axial plane using a standard bone algorithm at 0.6 mm. Imaging analysis was performed as described in the following paragraphs by investigators blinded to any clinical information or radiological diagnosis. All distance and length measurements were performed using ImageJ/Fiji software (version 1.52q^21^). 3D volumetric measurements of TB pneumatization were performed using the 3D Slicer software (version 4.10.2^22^).

### Anteroposterior and mediolateral TB dimensions

**Figure 1:**
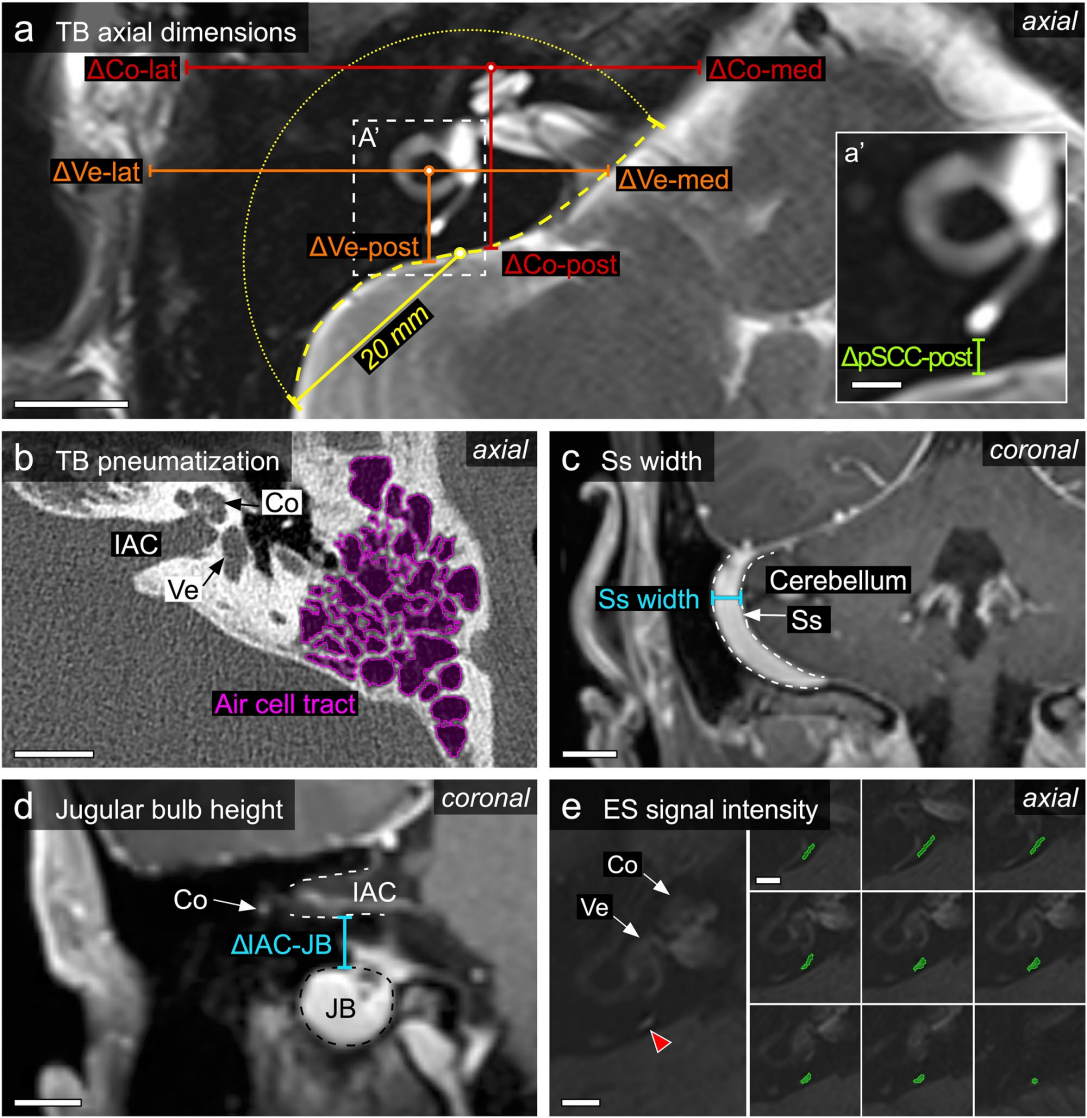
Summary of the TB parameters that were determined and the methods that were used. (**a**) Geometrical dimensions of TB (“bone thickness”) in the axial plane. ΔCo-lat/med/post: distance between the apex of the cochlea and the lateral, medial and posterior TB borders (red lines), respectively. ΔVe-lat/med/post: distance between the center of the horizontal semicircular canal and the lateral, medial and posterior TB borders (orange lines), respectively. To analyze the curvature of the posterior TB surface, a segment of the posterior TB border (yellow dashed line) within a circle (dotted yellow line) with a radius of 2 cm (yellow line) and its center at the exit of the vestibular aqueduct (yellow dot) was extracted. Scale bar 10 mm. (**a′**) ΔpSCC-post: distance between the most posterior edge of the posterior semicircular canal and the posterior TB surface. Scale bar 5 mm. (**b**) The pneumatized volume of the mastoid was determined by manually segmenting (outlined in magenta) and reconstructing its air cell tracts using HRCT imaging data. *Co* cochlea, *IAC* internal auditory canal, *Ve* vestibule. Scale bar 10 mm. (**c**) Sigmoid sinus (Ss; outlined by dashed white lines) width was measured at the level of the most lateral extension of the cerebellum. Scale bar 10 mm. (**d**) Height of the jugular bulb (JB) was determined by measuring its distance to the internal auditory canal. Co, cochlea. Scale bar 10 mm. (**e**) Inversion recovery SI of the ES (red arrowhead) was determined after manually segmenting the signal along its entire length (green outlines in serial image series). Scale bars 5 mm.

Length and width measurements were performed on Gd-MRI data (axial T2-weighted sequences; field of view of 220 mm, section thickness of 2 mm, matrix size 384 × 384). From the cochlear apex (measurement point ‘Co’) and the center of the horizontal semicircular canal (vestibular; measurement point ‘Ve’), the distances to the posterior (ΔCo-post, ΔVe-post), medial (ΔCo-med, ΔVe-med) and lateral (ΔCo-lat, ΔVe-lat) TB surfaces were determined (Fig. 1a). Moreover, the distance between the most posterior border of the posterior semicircular canal (pSCC) and the posterior TB surface (ΔpSCC-post) was determined (Fig. 1a′) in the radiological data, as well as in histologically processed TB specimens.

### Tortuosity of the posterior TB surface

The tortuosity of the posterior TB surface was analyzed in the axial plane at the level of the operculum (opening of the vestibular aqueduct into the posterior cranial fossa). A line segment of the posterior TB border with its length defined by a circle with its center at the operculum and a radius of 2 cm was extracted manually in Fiji^21^ using the “Export_multiset” macro^23^ (Fig. 1a). The tortuosity *T* was defined as the arc-chord ratio *T* = *L*/*D*, where *L* is the actual length of the line segment, and *D* is the straight-line distance between the endpoints of line segment. Tortuosity has a value of 1 for a straight line and > 1 for twisted lines. All numerical computations were performed in Python (Python Language Reference, version 3.6, Python Software Foundation, Wilmington, DE, USA).

### Volumetric quantification of TB pneumatization

In patients in whom dedicated HRCT imaging of the TBs was available, we quantified the volume of the TB air cell tracts, excluding the middle ear space and pneumatized spaces in the petrous apex, by manually segmenting the pneumatized areas on axial sections with a threshold range of − 1024 to 200 Hounsfield units (HU) for pneumatized areas and 600–3052 HU for bone (Fig. 1b). The respective volumes were reconstructed and calculated using the segmentation module and the segment statistics module in the 3D Slicer software.

### Sigmoid sinus (Ss) width and jugular bulb (JB) height

The width of the right and left Ss was measured along horizontal lines at the level of the most lateral extension of the cerebellar hemispheres in a coronal T2-weighted MR image (Fig. 1c). The distance between the most cranial extension of the JB and the floor of the internal auditory canal (IAC) was measured along a vertical line in a coronal T2-weighted MR image (Fig. 1d).

### Inversion recovery signal intensity of the ES within the vestibular aqueduct in Gd-MRI scans

In Gd-MRI data sets (3D inversion recovery sequence acquired 4 h post i.v. Gd injection) from MD-dg patients with unilateral MD (n = 40), we manually segmented the MR signal along the vestibular aqueduct from its proximal end near the vestibulum, corresponding to the location of the endolymphatic duct within the vestibular aqueduct, to its posterior end distal to the operculum, corresponding to the location of the extraosseous ES. Segmentations were performed in both TBs from each patient using the segment editor module in the 3D Slicer software (Fig. 1e). For each vestibular aqueduct, the mean signal intensity (mSI; brightness value in arbitrary units [a.u.]) was determined and normalized to the SI of the cerebrospinal fluid (CSF) signal in the IAC on the clinically non-affected side (normalized mSI [nmSI]). The ratio between the nmSI of the affected (nmSI_affected_) and the non-affected (nmSI_non-affected_) sides was determined (nmSI_affected_/nmSI_non-affected_).

### Statistical analysis

All statistical tests were selected before data collection. Each ear was analyzed as a separate experimental unit. Although the two ears of a same are never completely independent, treating ears as separate experimental units is justified by the fact that the two ears rarely match in their predisposition and clinical presentation in MD. To compare measurements among > 2 groups, one-way ANOVA was used. Following one-way ANOVA, pairs of means were compared, and the Holm–Sidak test was used to correct for multiple comparisons. All p values given are adjusted for multiple comparisons. Statistical analyses were performed using IBM SPSS Statistics for Windows (version 25, IBM Corp., Armonk, NY, USA) and Prism (version 7, GraphPad Software, La Jolla, CA, USA). The significance level was set to p < 0.05. If not otherwise specified, values are reported as the mean and standard deviation (SD) or as absolute number and percentage.

### Ethical approval

This study was approved by the local Ethics Committee (Kantonale Ethikkommission, Zurich, Switzerland, application number KEK-ZH-2016-01619). The archival human TB study was approved by the respective institutional review board (Mass General Brigham Human Research Committee, Boston, MA, USA, IRBNet-ID 880454-1). All parts of the study were performed in accordance with the relevant guidelines and regulations including the Helsinki declaration. Written informed general consent was obtained from all participants.

## Results

A total of 71 MD patients were included (one patient from the cohort was excluded due to missing imaging data that were required for the present analyses). Of those, 55 patients (77.5%) had previously been assigned to the MD-dg subgroup, and 16 patients (22.5%) had previously been assigned to the MD-hp subgroup^15^ (Table 1).

### Hypoplastic posterior (retrolabyrinthine) TB portion in the MD-hp group

**Figure 2:**
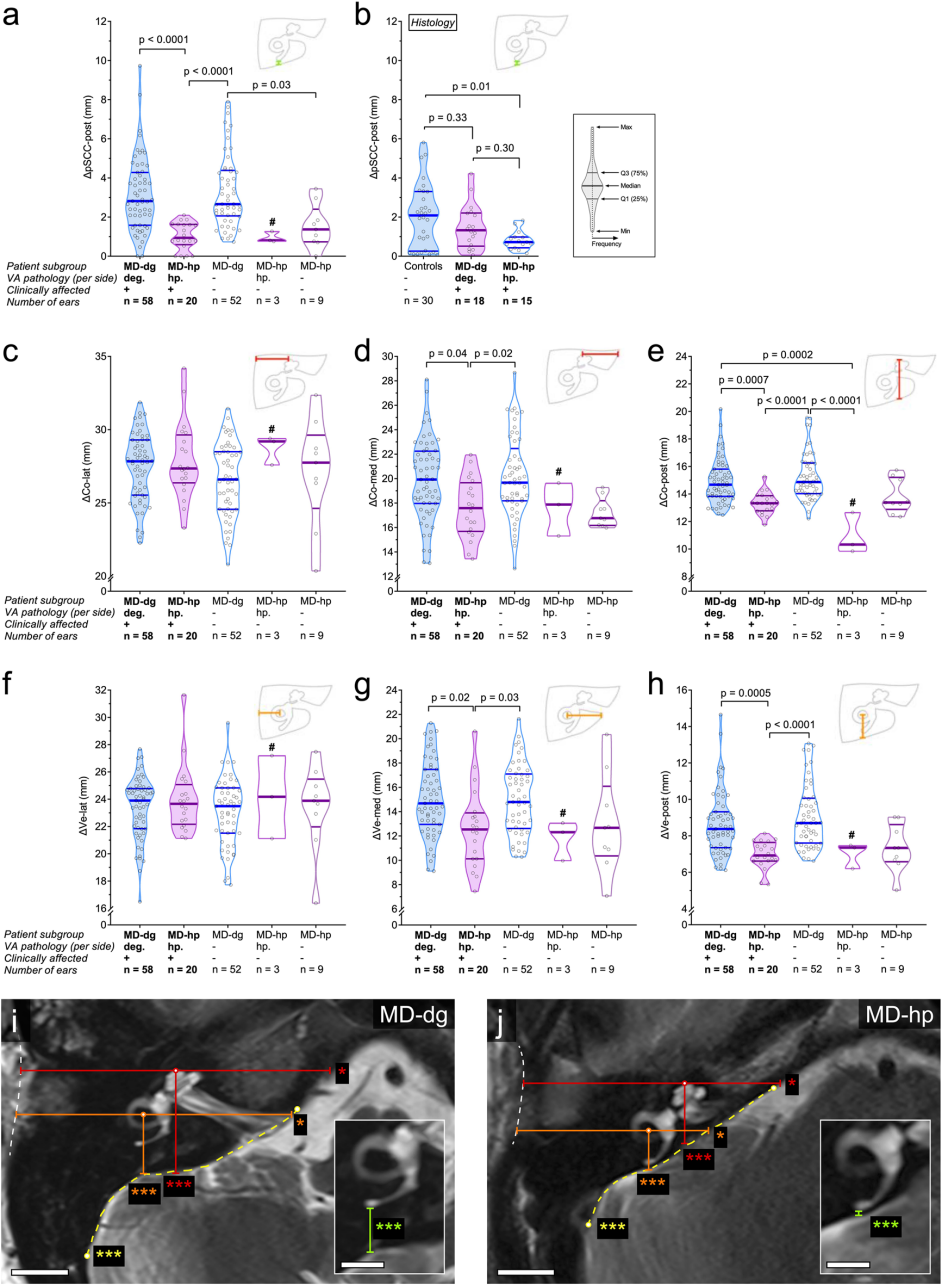
TB geometrical dimensions in the axial plane. Each data point represents one ear. In total, five groups of ears were analyzed for each radiologic feature. The first and second group were affected ears of MD-dg (n = 58) and MD-hp patients (n = 20), respectively. The third group were unaffected ears of MD-dg patients (n = 52). Unaffected ears of MD-hp patients were split into two groups according to whether they also exhibited a hypoplastic VA pathology (fourth group, n = 3) or not (fifth group, n = 9). (**a**,**b**) Thickness of the bony covering between the posterior semicircular canal and the posterior TB surface (ΔpSCC-post), as measured in MRI data (**a**) and histologic TB specimens (**b**) (green lines in (**i**) and (**h**)). (**c–e**) Distance from the apex of the cochlea to the lateral (ΔCo-lat; **c**), medial (ΔCo-med; **d**) and posterior (ΔCo-post; **e**) TB borders (red lines in (**i**,**j**)). (**f–h**) Distance from the center of the horizontal semicircular canal (vestibule; Ve) to the lateral (ΔVe-lat; **f**), medial (ΔVe-med; **g**) and posterior (ΔVe-post; **h**) TB borders (orange lines in (**i**,**j**)). (**i–j**) Representative TB geometry (MRI) in the MD-dg group (**i**) and the MD-hp group (**j**). Asterisks indicate significantly different axial dimensions between the MD-dg and MD-hp groups. *p < 0.05, ***p < 0.001 (exact p values are provided in **a**–**h**). ^#^Group excluded from statistical analysis due to the low number of observations. Scale bars 10 mm; inset scale bars 5 mm.

Measurements of TB geometrical features (Figs. 1a, 2a–j) demonstrated significant intergroup (MD-hp vs. MD-dg) differences for ΔCo-post (MD-dg: 14.9 [1.6] mm; MD-hp: 13.4 [0.8] mm; p = 0.0007; Fig. 2e), ΔVe-post (MD-dg: 8.7 [1.7] mm; MD-hp: 7.0 [0.8] mm; p = 0.0005; Fig. 2h), ΔCo-med (MD-dg: 19.9 [3.3] mm; MD-hp: 17.6 [2.4] mm; p = 0.04; Fig. 2d), ΔVe-med (MD-dg: 15.0 [3.0] mm; MD-hp: 12.6 [3.3] mm; p = 0.02; Fig. 2g), and ΔpSCC-post values (MD-dg: 3.1 [1.9] mm; MD-hp: 12.6 [3.3.3] mm; p = 0.02; Fig. 2a). Histological TB specimens also showed a trend toward a shorter ΔpSCC-post values in the MD-hp group than in the MD-dg group (MD-dg: 1.5 [1.1] mm; MD-hp: 0.8 [0.5] mm; p = 0.30; Fig. 2b); however, no specimen exhibited true dehiscence of the pSCC. Exemplary MR images of all TB geometrical features are shown in an MD-hp patient (Fig. 2i) and in an MD-dg patient (Fig. 2j).

**Figure 3:**
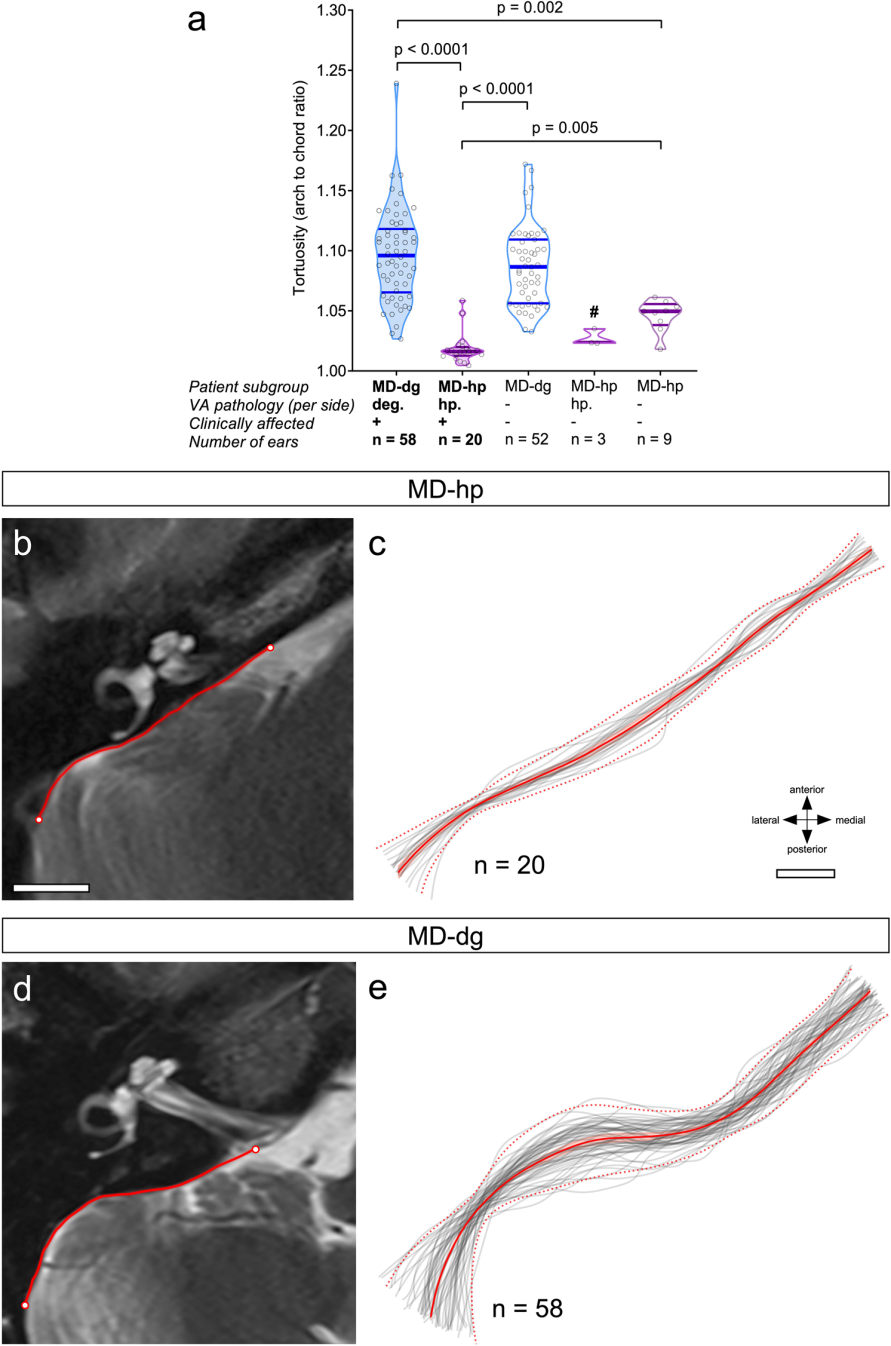
Two-dimensional shape of the posterior TB surface. (**a**) Tortuosity of the posterior TB surface in the axial plane at the level of the operculum. ^#^Group excluded from statistical analysis due to the low number of observations. (**b–e**) Red lines in (**b**) and (**d**) are outlines of the posterior TB surface (see methods section and yellow dashed lines in Figs. 1a, 2i,j), red continuous lines in (**c**,**e**) represent the means from all extracted TB outlines (grey lines) and red dashed lines represent two standard deviations in each direction from the mean for the MD-hp group (**c**) and the MD-dg group (**e**). The extracted line segments from all included TBs were resampled using B-splines so that they consisted of 1000 sample points each and were aligned spatially using Procrustes analysis^24^. Scale bar 10 mm.

Intergroup comparison of the two-dimensional shape of the posterior TB surface (Fig. 1a) demonstrated a significantly lower line curvature (arch-to-chord ratio), i.e., a flatter posterior TB surface in the MD-hp group than in the MD-dg group (MD-dg: 1.096 [0.038]; MD-hp: 1.019 [0.013]; p < 0.0001; Fig. 3). Exemplary outlines of the posterior TB surface in an MD-dg and an MD-hp patient are depicted in Fig. 2i–j (yellow broken lines).

### Hypoplastic mastoid air cell tracts in the MD-hp group

**Figure 4:**
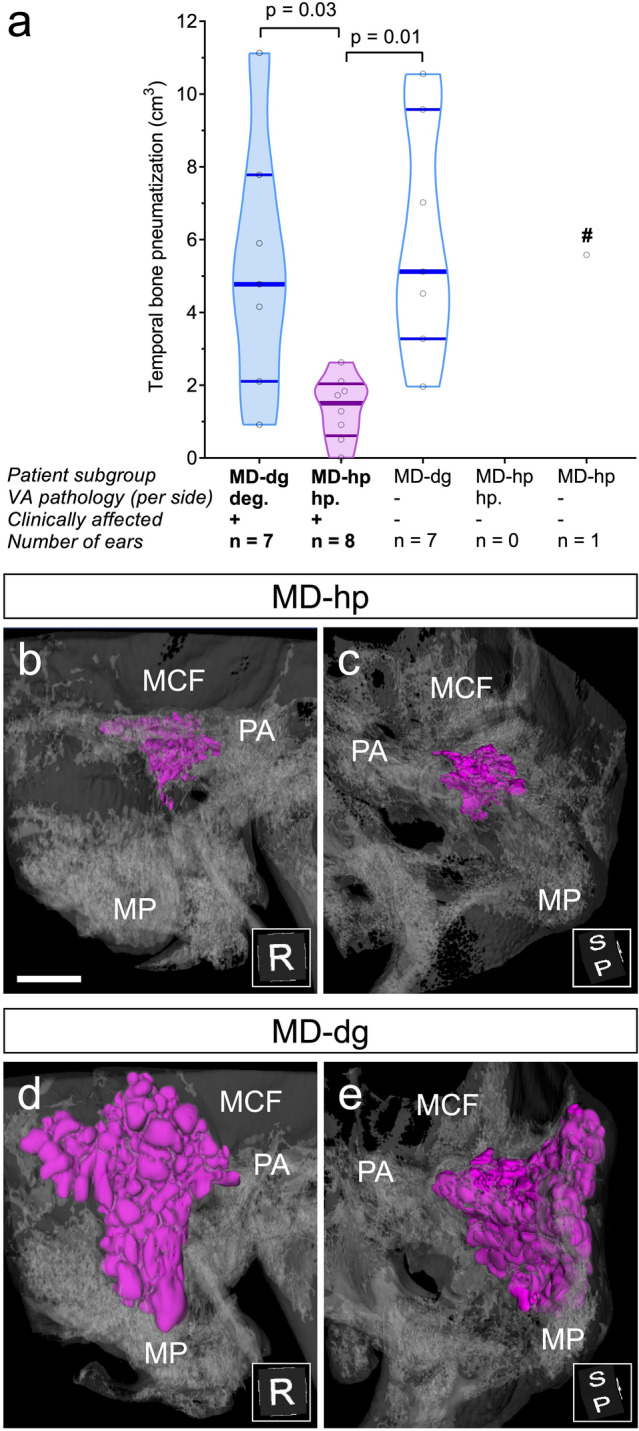
TB Pneumatization. (**a**) Pneumatized TB volume. ^#^Data point excluded from statistical analysis. (**b–e**) Reconstruction of the segmented pneumatized TB volume (magenta) in representative MD-dg (**b**,**c**) and MD-hp (**d**,**e**) patients. Bone is shown in transparent gray. *MCF* middle cranial fossa, *MP* mastoid process, *PA* petrous apex. Insets show 3D orientation. *P* posterior, *R* right, *S* superior. Scale bar 10 mm.

A total of 23 TB HRCT data sets from 12 patients were included in the analysis. One TB HRCT data set was excluded due to an anterior mastoidectomy that was performed for cochlear implantation. Although carried out in a subsample of the present study, an intergroup comparison of the total pneumatization volume (Fig. 1b) in the affected ears showed a significantly smaller volume in the MD-hp group than in the MD-dg group (MD-hp 1.37 [0.86] cm^3^; MD-dg: 5.25 [3.45] cm^3^; p = 0.03, Fig. 4a). Within the MD-dg group, the volume between the affected and the non-affected side did not differ significantly (MD-dg affected 5.25 [3.45] cm^3^; MD-dg non-affected 6.00 [3.20] cm^3^; p = 0.94). Notably, a statistical intergroup comparison of pneumatization volumes between clinically unaffected ears was not possible due to the small number of individuals in the MD-hp group with unilateral MD (n = 1). Exemplary HRCT-based volume reconstructions of the segmented air cell tracts in an MD-hp patient (Fig. 4b,c) and in an MD-dg patient (Fig. 4d,e) are shown.

### Reduced Ss width and JB height in affected ears in the MD-dg group

**Figure 5:**
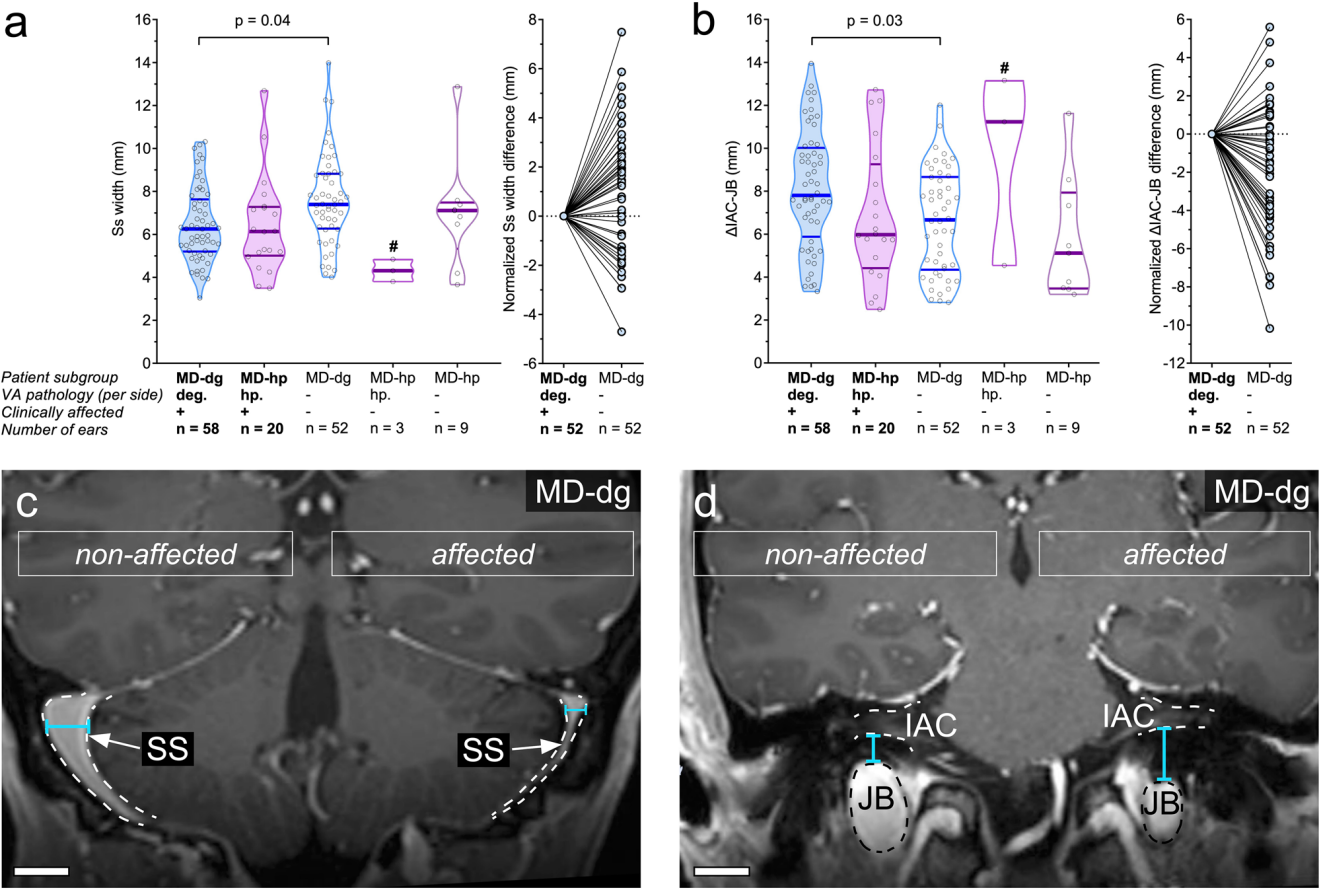
Sigmoid sinus (Ss) width and jugular bulb height (ΔIAC-JB). (**a**,**b**) Ss width (**a**) and ΔIAC-JB (**b**) among the MD-dg and MD-hp patient groups. The corresponding line charts on the right show paired and normalized (affected side = 0 mm) Ss width (**a**) and ΔIAC-JB (**b**) in MD-dg patients with unilateral MD (n = 52). Larger Ss widths/ΔIAC-JB on the unaffected side yield positive values on the y axis. Lines connect the affected and unaffected ears of individual patients. ^#^Group excluded from statistical analysis. (**c**,**d**) Exemplary MR images of typical Ss width and ΔIAC-JB in an MD-dg patient affected by left unilateral MD. Scale bars 10 mm.

Intergroup comparison of the Ss width, as measured at the level of the most lateral extension of the cerebellar hemispheres in the coronal imaging plane (Fig. 1c), demonstrated no significant difference between groups (MD-dg: 6.45 [1.74] mm; MD-hp 6.41 [2.29] mm; p = 0.94; Fig. 5a). Within the MD-dg group, the Ss width on the clinically affected side was significantly smaller than that on the non-affected side (affected side: 6.5 [1.7] mm; non-affected side: 7.6 [2.1] mm; p = 0.04; Fig. 5a). Intergroup comparison of the maximum height of the JB, as measured by the distance between the cranial border of the JB and the floor of the IAC in the coronal imaging plane (Fig. 1d), demonstrated no significant difference (MD-dg: 8.13 [2.75] mm; MD-hp: 6.85 [3.16] mm; p = 0.37; Fig. 5b). Within the MD-dg group, ΔIAC-JB was significantly longer on the clinically affected side (affected side: 8.1 [2.7] mm; non-affected side: 6.6 [2.4] mm; p = 0.03; Fig. 5b). Exemplary MR images of MD-dg patients with unilateral MD, reduced Ss measurements (Ss width; Fig. 5c) and reduced JB vessel calibers (ΔIAC-JB; Fig. 5d) on the clinically affected side are shown.

### Reduced inversion recovery SI of the distal ES in clinically affected ears of the MD-dg group

**Figure 6:**
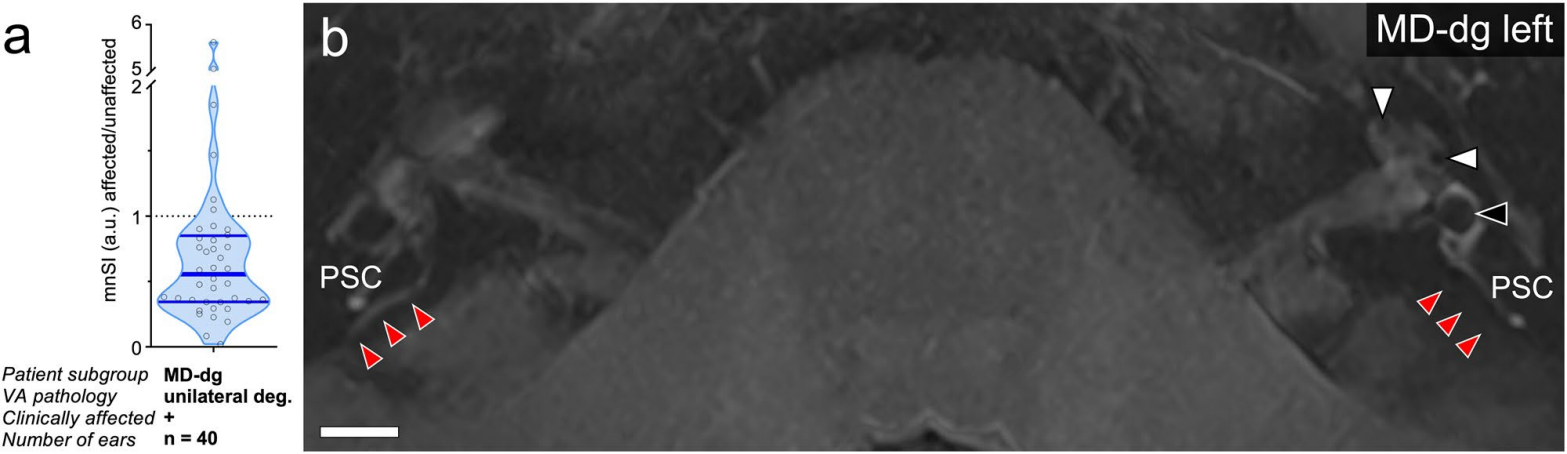
ES signal intensity in unilateral MD-dg cases. (**a**) The ratio of the normalized mean signal intensity (nmSI) between the affected and unaffected sides was determined after segmenting the ES signal (Fig. 1e) in axial MR images (3D IR sequence, maximal intensity projection). (**b**) Representative MD-dg patient with a clinical diagnosis of left MD and endolymphatic hydrops in the left cochlea (white arrowheads) and saccule (black arrowhead). Red arrowheads indicate the ES signal with its most distal (posterior) extension. *PSC* posterior semicircular canal. Scale bar 10 mm.

The mean nmSI_affected_/nmSI_non-affected_ ratio from all MD-dg patients with unilateral MD was determined to be 0.56 (interquartile range [IQR] 0.34–0.85). A ratio < 1, indicating a relative decrease in SI on the clinically affected side compared to the non-affected side (Fig. 6), was found in 36 (90%) of MD-dg patients. The most pronounced and consistent decrease in inversion recovery SI was overall observed in the most-posterior TB region near the operculum, which corresponds to the location of the extraosseous ES (red arrows in Fig. 6).

## Discussion

For many of the radiological TB features (re)investigated in the present study, inconclusive^6^ or contradictory^25,26^ prevalence data were reported previously in MD patients. Here, we instead distinguished two etiopathologically distinct patient groups, the MD-hp group (~ 30% of all MD patients), and the MD-dg group (~ 70% of all MD patients), characterized by developmental hypoplasia and degeneration of the ES, respectively^12,15^. We demonstrated several of the examined radiological TB features to be predominantly prevalent in either of the two patient groups. Furthermore, the most TB features appeared to be linked to the respective group’s etiopathogenic context—i.e. developmental vs. degenerative.

In the MD-hp group, the pathognomonic histopathology of ES hypoplasia features a lower-than-normal number of ES epithelial cells, and the remaining epithelium features diminished protein expression of ion channels and transporters^12^. Impaired local ion transport in the hypoplastic ES most likely has propagating disrupting effects on ion and fluid homeostasis throughout the inner ear. Thereby, ES hypoplasia presumably critically contributes to the pathogenesis of endolymphatic hydrops and MD^12,27^. The etiology of ES hypoplasia remains unknown. Its morphological resemblance with the fetal/newborn human ES^28^ points toward a premature developmental arrest^13,29^. The invariably associated hypoplastic bony vestibular aqueduct, which takes an abnormally straight and short course through the posterior TB portion (see material and methods section, diagnostic criteria for the MD-hp group^13^), further indicates a developmental TB pathology. Here, we identified additional hypoplastic TB features, including poorly pneumatized air cell tracts and a thinned, abnormally flat posterior TB surface, in the MD-hp group. The combined presence of all hypoplastic features in the retrolabyrinthine bone suggests a regional developmental defect, possibly due to a distinct genetic etiology that remains to be investigated. We suppose that the hypoplastic bone features are merely epiphenomena, whereas ES hypoplasia (loss of ES function) is actually relevant in the pathogenesis of clinical MD symptoms^30^. Notably, the retrolabyrinthine bony layer, upon radiological analysis, appeared thinned to the point of a suspected dehiscence of the pSCC (ΔpSCC-post = 0 mm) in a considerable number of MD-hp patients, although histological analysis in a small sample of postmortem MD-hp and MD-dg cases (n = 32), did not demonstrate any signs of “true” pSCC dehiscence. We here found a ΔpSCC-post < 2 mm to be exclusive to the MD-hp group, meaning a value > 2 mm virtually excluded the MD-hp endotype and may thus be used as an additional radiological marker in radiological endotyping, in addition to the angular trajectory of the vestibular aqueduct^13^.

In the MD-dg group, the pathognomonic histopathology of ES degeneration features deteriorating ES epithelial cells and local fibrotic remodeling. The resulting loss of epithelial ion channels and transporters^12^ probably has the same pathophysiological impact on inner ear fluid homeostasis in MD as presumed for ES hypoplasia (see previous paragraph^12,27^). The underlying cause of ES degeneration is unknown. One possible pathomechanism that could primarily and selectively damage the ES epithelium is ischemic injury. The microvascular supply of the ES is terminal and is largely separate from that of the cochlea and the vestibular labyrinth (no or few anastomoses)^31–33^, and thus likely predisposes for local ischemic events. Hypotheses on a vascular compromise to the inner ear as a causal factor in MD were previously considered. Radiology studies investigated vascular anomalies in the TB region and compared the course and caliber of major blood vessels between MD patients and control cohorts^34–36^; mostly though with variable/ambiguous/inconclusive data among studies. Here, we re-assessed radiological parameters (course and caliber, respectively) of the Ss and the JB, both of which directly receive blood from inner ear venules^37^ and are thus part of the inner ear venous drainage system. We found a significantly decreased cross-sectional diameter (smaller caliber) of the Ss and a significantly increased distance between the JB and the IAC (divergent course) in clinically affected TBs as compared to non-affected TBs in the MD-dg group. However, both features were highly variably expressed with no clear-cut distinction (standard deviation errors overlapping between groups) between clinically affected and non-affected TBs from MD-dg. Whether a smaller caliber of the Ss or the divergent course of the JB affect the venous drainage from the inner ear in patterns that cause selective ischemia and degeneration of the ES is not known. Notably, side asymmetries in the caliber and course of these venous vessels are well known and widespread norm variants^38^. Together, evidence for the presence of vascular anomalies in major vessels of the inner ear’s venous drainage system in the MD-dg group is inconclusive. Other pathomechanisms, such as e. g. local inflammatory injury^39,40^, may—at least in some members of the MD-dg group—better explain the degenerative histopathology of the ES.

Upon further analysis, we almost invariably detected a relative decrease in the inversion recovery Gd-MRI signal that originated from the ES in the clinically affected inner ears of MD-dg patients. The focal SI loss was most pronounced in the distal (extraosseous) ES, which is also the ES portion that is most severely affected by epithelial degeneration^12^. Thus, the inversion recovery Gd-MRI signal loss may be a radiological correlate of this histopathology. In contrast, in four MD-dg patients, we found a 1.5-fold or higher inversion recovery Gd-MRI signal from the ES on the clinically affected side compared to that on the non-affected side. Focal signal hyperintensity of the ES upon MRI-Gd imaging was already associated with a broad range of cochleovestibular symptoms (not with MD) and was interpreted as active inflammation of the ES (“endolymphatic sacitis”^40^). For the present four cases, however, the available clinical records did not indicate an early disease stage and did not mention any acute disease activity at the time of MRI data acquisition, which impedes any interpretation of their disparate MRI findings.

## Conclusion

Expressional variability of radiological TB features among MD patients can be explained by the existence of, at least two, different disease endotypes. Future studies investigating the etiologies of MD, instead of including patients solely based on the clinical diagnosis of MD, may consider patient (sub)groups with genetically, pathologically, or clinically distinct endotypes to gather more meaningful data.

## Data Availability

The datasets generated and analyzed during the current study are available from the corresponding author on reasonable request.

## Abbreviations

ΔCo-med: Distance from the cochlea to the medial TB surface
ΔCo-lat: Distance from the cochlea to the lateral TB surface
ΔCo-post: Distance from the cochlea to the posterior TB surface
ΔVe-med: Distance from the center of the hSCC to the medial TB surface
ΔVe-lat: Distance from the center of the hSCC to the lateral TB surface
ΔVe-post: Distance from the center of the hSCC to the posterior TB surface
ΔpSCC-post: Distance from the pSCC to the posterior TB surface
ΔIAC-JB: Distance from the internal auditory canal to the jugular bulb
ES: Endolymphatic sac
hSCC: Horizontal semicircular canal
MD: Meniere’s disease
pSCC: Posterior semicircular canal
Ss width: Horizontal width of the sigmoid sinus
TB: Temporal bone
